# A Case of Dog Bite Identification Using Trace DNA Recovered from Clothing Without Apparent Bite Marks

**DOI:** 10.3390/ani15243587

**Published:** 2025-12-14

**Authors:** Reina Ueda, Yuko Kihara, Aki Tanaka

**Affiliations:** Laboratory of Wildlife Medicine, Department of Veterinary Medicine, Nippon Veterinary and Life Science University, 1-7-1 Kyonan-cho, Musashino-shi 180-8602, Tokyo, Japan

**Keywords:** forensic veterinary medicine, STR, individual identification, dog bite incident

## Abstract

Dog bite incidents represent a significant public health concern, and DNA analysis, in addition to morphological comparison, has been recognized as a useful tool for identifying the offending dog. STR typing has been established as a reliable method for individual identification in dogs, with many successful applications reported. However, most previous studies have analyzed samples showing visible traces such as blood or saliva, and there have been no documented cases in which the offending dog was identified from clothing without apparent evidence. In the present study, STR analysis was performed on trace DNA extracted from the clothing of a victim who sustained a minor dog bite injury. The STR profile obtained from the sample completely matched that of one of the three suspected dogs, a Weimaraner, at all 19 loci examined. No visible saliva or blood contamination was observed on the clothing. This case demonstrates that even trace DNA from clothing without visible markings can yield a complete STR profile when appropriate sampling and analytical methods are applied. The findings highlight the potential of canine DNA forensics to contribute to animal-related investigations and underscore the importance of accumulating allele frequency data and standardizing analytical procedures for future applications.

## Simple Summary

Dog bite incidents represent a public health concern, and DNA analysis, including short tandem repeat (STR) typing, has been utilized as an effective method for identifying offending dogs. Previous reports of DNA analysis using the victim’s clothing primarily involved samples with visible contamination by blood or saliva, or with apparent damage to the fabric. In the present study, we analyzed trace amounts of DNA recovered from clothing that showed no visible marks or contamination. As a result, an STR profile completely matching that of one of three suspected dogs was obtained. This finding demonstrates a rare case in which the individual identification of the offending animal was achieved through appropriate DNA analysis, even from clothing with no observable evidence.

## 1. Introduction

Dogs are among the most familiar companion animals in human society; however, dog bites and attacks occur frequently and constitute a major public health concern [1,2]. Such incidents range from minor bite injuries to severe or even fatal trauma and have drawn considerable social attention [2,3,4]. Many of these cases involve vulnerable populations, including the elderly and young children [5].

Forensic investigations of dog bite incidents have traditionally employed morphological comparisons of wound characteristics and tooth impressions, as well as DNA analyses of materials derived from the suspected dog [6]. Among the available molecular approaches, STR analysis is widely used for individual identification in dogs. Multiplex PCR systems specifically designed for canine STR typing possess high specificity and sensitivity comparable to commercially available human STR kits [2,6]. Moreover, canine STR profiling has been shown to yield reliable results even in the presence of mixed human DNA, making it a valuable tool for identifying offending dogs from saliva or blood collected from the victim’s wounds or clothing [7,8,9].

Most previous forensic reports of canine STR analysis from human clothing have been associated with severe or fatal cases, where visible traces such as saliva stains, fabric damage, blood, or hair were present [8,9,10]. In contrast, there have been almost no reports of successful identification of the offending dog using only clothing samples that lack visible contamination or damage.

The present study describes a forensic case in which STR analysis was conducted on trace DNA obtained from the clothing of the victim who sustained a dog bite injury. This case provides a rare example demonstrating that reliable DNA profiles can be obtained from visually unmarked materials and highlights the potential application of DNA forensic techniques in a wide range of animal-related incidents.

## 2. Case

In 2025, a young girl sustained a minor injury after being bitten by a dog kept and managed at a private facility. Because negligence in the management of the animal was suspected at the facility, a criminal investigation was initiated under the charge of professional negligence resulting in injury. Subsequently, forensic investigators submitted to our laboratory a pair of trousers worn by the victim at the time of the incident, along with three FTA cards containing saliva samples collected from three dogs kept at the same facility, a Weimaraner, a Standard Poodle, and a Bernedoodle, as reference samples. At the request of the police authorities, an individual identification was conducted to compare and discriminate between the DNA profiles obtained from the trousers and those of the three suspected dogs, in order to determine the offending animal.

## 3. DNA Analysis

### 3.1. Sampling and DNA Extraction

**Figure 1:**
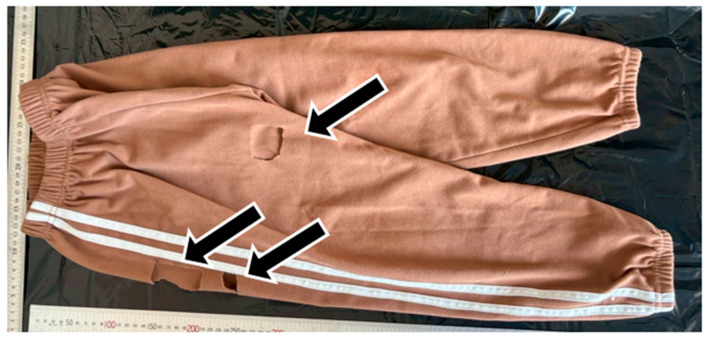
Sampling area excised from the trousers for DNA analysis in this study.

**Figure 2:**
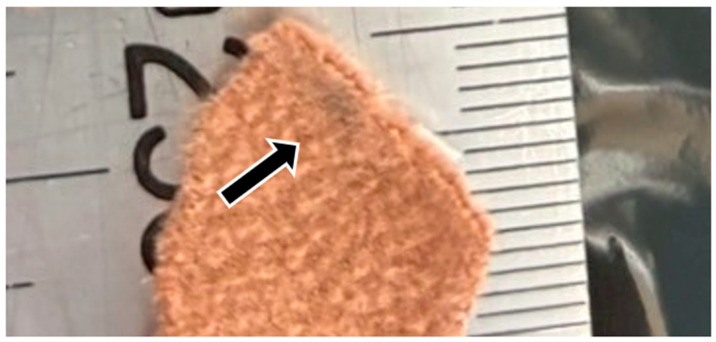
Enlarged view of the discolored area on the fabric sample excised from the trousers in this study.

DNA analysis was conducted on one clothing sample (the trousers worn by the victim) and three reference samples consisting of FTA cards bearing saliva from the suspected dogs. All materials were stored at −30 °C from the time of submission until DNA extraction to prevent degradation. Based on photographic evidence provided by the police, the section of the trousers corresponding to the presumed bite area was identified and excised for analysis (Figure 1). The fabric from the suspected bite region was used as an extraction sample. Visual inspection of the material revealed no evidence of physical damage, blood, or hair adhering to the fabric surface. Only a small, slightly discolored area approximately 0.2 cm in diameter was noted within the region corresponding to the injury (Figure 2). From the outer surface of the trousers, a fabric piece measuring 3 cm (vertical) × 5 cm (horizontal) was collected from a location 5 cm to the right side of the crotch area. In addition, a section measuring 6 cm (vertical) × 3–4 cm (horizontal) was excised from a position approximately 15.5 cm below the right waist. Furthermore, the discolored area, approximately 0.2 cm in diameter, was visually identified immediately below this section, and a fabric piece measuring 1.5 cm (vertical) × 2 cm (horizontal) that included this discolored region was excised.

The excised trouser sample was processed using the QIAamp DNA Investigator Kit (QIAGEN, Hilden, Germany). Because the amount of saliva residue on the fabric was presumed to be minimal, carrier RNA [11] was added during extraction to enhance DNA recovery efficiency. The isolated DNA was eluted in 50 µL of Buffer ATE. For the three FTA card samples, approximately three discs (each about 3 mm in diameter) were punched from each card and processed using the same kit. The final elution volume for each was adjusted to 50 µL. All extracted DNA solutions were stored at 2–5 °C until subsequent analysis.

All samples were processed, and DNA extraction was completed within four days after the specimens were delivered to our laboratory. Subsequent analyses were also carried out without delay, and the entire set of examinations was completed within approximately two weeks.

### 3.2. PCR Amplification and Fragment Analysis

To determine the DNA profiles for comparison between the DNA extracted from the trousers and those of the three suspected dogs, individual identification was performed using a commercially available canine STR typing kit, the Thermo Scientific Canine Genotypes Panel 2.1 (Thermo Fisher Scientific, Waltham, MA, USA). This kit enables the analysis of 18 autosomal STR loci and one sex-determining marker (amelogenin), as summarized in Table 1. A blank sample was included in each PCR reaction as a negative control, and testing was conducted only after confirming that no amplification was observed at any locus.

PCR amplification was performed by multiplex co-amplification according to the manufacturer’s protocol for the Thermo Scientific Canine Genotypes Panel 2.1. A T100 Thermal Cycler (Bio-Rad, Hercules, CA, USA) was used for amplification. Fragment analysis was outsourced to Fasmac Co., Ltd. (Kanagawa, Japan), using 500 LIZ as the size standard and filter set G5. The resulting electropherograms were manually genotyped using Peak Scanner Software v2.0 (Thermo Fisher Scientific, Waltham, MA, USA), and allele binning was performed to assign integer values.

In a previous study involving 150 dogs representing several breeds (Golden Retriever, Miniature Dachshund, and Shiba Inu), the random match probability obtained using the Thermo Scientific Canine Genotypes Panel 2.1 was reported to be extremely low, 3.257 × 10^−16^, 3.933 × 10^−18^, and 2.107 × 10^−18^, respectively [12]. In the present case, the breeds analyzed were Weimaraner, Standard Poodle, and Bernedoodle. Although allele frequency distributions may vary among breeds, these findings suggest that the probability of random matching is exceedingly small, estimated to be in the range of one in several quadrillions, thus allowing for reliable individual identification.

In this study, we conducted the analyses with full consideration of the potential occurrence of various artifacts, including allele dropout, false allele, and peak imbalance. For allele calling from STR electrophoresis data, each peak observed within the expected size range was carefully inspected visually, and its peak morphology and noise characteristics were evaluated in comparison with our previous casework and published findings. Based on these assessments, we comprehensively judged the possible impact of the observed genotypes on individualization and confirmed the validity of the results obtained in this case.

## 4. Result

Complete DNA profiles were successfully obtained from all four samples, consisting of the trousers and the saliva samples from the three suspected dogs. After allele binning, considering an acceptable inter-run variation of ±0.1–0.4 bp (corresponding to a 1 bp difference after normalization), the canine DNA profile obtained from the trousers was found to be completely identical to that of Candidate 1 (Weimaraner), the dog suspected of having inflicted the bite (Table 2). STR electropherograms are included in the Supplementary Materials.

## 5. Discussion

In the present case, a complete canine DNA profile enabling individual identification of the offending dog was successfully obtained from the victim’s clothing, despite the absence of any visible biological traces. Conventionally, forensic examinations in animal bite investigations target samples that contain clearly identifiable biological materials such as blood, hair, or tissue fragments. This case represents a rare instance demonstrating that STR-based DNA profiling can be achieved even from samples showing no apparent contamination or damage.

The success of DNA profiling in this case is presumed to have been influenced by the direct extraction of DNA from the fabric, without the use of adhesive tape or swabs, and the addition of carrier RNA during the extraction process, which likely enhanced DNA recovery efficiency. Relatively short analytical timeline likely minimized the effects of DNA degradation during storage and contributed to the favorable results obtained in this study. In addition, the reference photographs provided by the police, indicating the approximate location of the bite site, played a crucial role in identifying the sampling area. However, as the photographic reference only indicated the general area, more detailed and quantitative documentation, such as centimeter-scale localization of bite marks, would be desirable in future investigations. Such improvements could further enhance the accuracy, reliability, and reproducibility of DNA detection from trace or visually unmarked evidence. Examiners should also carefully observe the entire sample, including regions beyond the estimated bite area, to detect any remaining biological traces from the involved animal.

Because allele frequency data for each of the three dog breeds involved in this study were limited, the calculated random match probabilities should be regarded as estimates and may not fully reflect breed-specific genetic structures. This represents one of the limitations of our study. In the future, as breed-specific databases become more established, it will be possible to evaluate random match probabilities with greater accuracy.

Dog bite incidents such as this often result from inappropriate management by owners or caretakers. Improper animal care not only constitutes a public safety issue but also represents a concern from the standpoint of animal welfare and may even escalate to abuse. Therefore, scientific verification in animal-related injury cases has social significance beyond the identification of the offending individual; it contributes to the prevention of recurrence and the promotion of responsible animal management practices. Moreover, dogs may not only act as offenders but also as victims or as sources of crucial forensic evidence in other criminal cases. As one of the most common companion animals, dogs frequently leave biological traces such as hair, saliva, blood, or feces at crime scenes. These materials can be secondarily transferred between suspects and victims during an incident. Previous reports have shown that canine DNA analysis has been effectively applied not only in cases of animal abuse or theft but also as evidence establishing links among suspects, crime scenes, and victims [9]. Taken together, this case highlights the scientific utility of animal-derived DNA analysis and underscores its potential contribution to the advancement of forensic veterinary medicine and human forensic sciences. Such analytical approaches may play an increasingly important role in elucidating animal-related cases and preventing their recurrence.

In this case, due to the limited amount of DNA available for analysis, genotypes were determined based on the results obtained from a single round of PCR amplification. However, single-round PCR carries the risk of allelic dropout, which can affect genotype interpretation, particularly in heterozygous loci where only one allele may be detected. In this study, we interpreted the genotypes with this potential risk. In future cases where sufficient amounts of DNA can be obtained, incorporating multiple PCR amplifications is expected to reduce the impact of allelic dropout and improve the reliability of genotype determination.

## 6. Conclusions

This report presents a rare case in which an STR profile enabling identification of the offending dog was successfully obtained from the victim’s clothing, despite the absence of any visible biological traces or fabric damage. The findings demonstrate that, even in samples showing minimal macroscopic evidence, appropriate sampling and analytical procedures can enable reliable identification of the offending animal. Canine DNA analysis has broad applicability—not only for investigating dog bite incidents but also for providing scientific evidence in cases of animal abuse and in human criminal investigations involving animal-derived materials. This case highlights the scientific utility of animal DNA analysis and suggests its potential contribution to the advancement of forensic veterinary and human forensic sciences, as well as to efforts aimed at case resolution and prevention of recurrence.

## Figures and Tables

**Table 1:** STR markers and sex-determining markers used in the analysis kit.

Locus	Chromosome	Repeat Motif	Size Range (bp)	Dye Color
PEZ02	17	GGAA	104–145	6FAM
ZFX/Y	X/Y	NA	159–164	6FAM
PEZ17	4	GAAA	190–225	6FAM
FH2017	15	AGGT_(m)_ AGAT_(n)_ GATA_(o)_	256–276	6FAM
FH2309	1	GAAA	339–428	6FAM
PEZ05	12	TTTA	92–117	VIC
FH2001	23	GATA	118–160	VIC
FH2328	33	GAAA	171–213	VIC
FH2004	11	AAAG	232–326	VIC
FH2361	29	GAAA	322–439	VIC
PEZ21	2	AAAT	83–103	NED
FH2054	12	GATA	139–177	NED
FH3377	3	GAAAA	183–305	NED
FH2107	3	GAAA	291–426	NED
FH2088	15	(TTTA)_m_ (TTCA)_n_	94–138	PET
vWF.X	27	AGGAAT	151–187	PET
FH2010	24	ATGA	221–243	PET
PEZ16	27	GAAA	280–332	PET
FH3313	19	GAAA	340–446	PET

**Table 2:** Comparison of STR profiles obtained from the trousers and saliva samples of the three suspected dogs.

Locus	Sample Collected from Trousers	Saliva Collected from Candidate 1	Saliva Collected from Candidate 2	Saliva Collected from Candidate 3
PEZ02	127, 127	127, 127	123, 127	122, 131
ZFX/Y	161, 165	161, 165	161, 165	161, 165
PEZ17	202, 210	202, 210	202, 210	215, 219
FH2017	265, 265	265, 265	265, 265	265, 265
FH2309	349, 399	349, 399	383, 395	391, 395
PEZ05	103, 103	103, 103	103, 103	103, 103
FH2001	129, 146	129, 147	129, 142	129, 147
FH2328	191, 191	191, 191	175, 198	202, 202
FH2004	239, 243	239, 244	243, 289	239, 239
FH2361	345, 408	345, 408	349, 408	345, 408
PEZ21	89, 89	89, 89	89, 97	97, 97
FH2054	150, 170	150, 170	154, 169	158, 168
FH3377	194, 239	194, 239	218, 239	204, 218
FH2107	368, 372	368, 371	371, 424	379, 424
FH2088	128, 132	128, 132	120, 128	128, 132
vWF.X	164, 164	164, 164	158, 164	164, 170
FH2010	239, 239	239, 239	231, 235	231, 235
PEZ16	292, 296	292, 296	308, 308	292, 292
FH3313	374, 396	374, 395	370, 443	410, 439

## Data Availability

This article contains all data found in this study. Further details or information can be directed to the corresponding author.

## Supplementary Materials

The following supporting information can be downloaded at: https://www.mdpi.com/article/10.3390/ani15243587/s1, STR electrophoresis of the trouser sample.

## Abbreviations

The following abbreviations are used in this manuscript:

STR	Short tandem repeat
PCR	Polymerase chain reaction

## References

[1] Salem N.H., Belhadj M., Aissaoui A., Mesrati M.A., Chadly A. (2013). Multidisciplinary approach to fatal dog attacks: A forensic case study. J. Forensic Leg. Med..

[2] Giovannini E., Roccaro M., Peli A., Bianchini S., Bini C., Pelotti S., Fais P. (2023). Medico-legal implications of dog bite injuries: A systematic review. Forensic Sci. Int..

[3] Di Donato S., Ricci P., Panarese F., Turillazzi E. (2006). Cane Corso attack: Two fatal cases. Forensic Sci. Med. Pathol..

[4] Di Nunzio M., Della Valle A., Serino A., Corrado F., Di Nunzio C. (2024). How the forensic multidisciplinary approach can solve a fatal dog pack attack. Forensic Sci. Med. Pathol..

[5] Sarenbo S., Svensson P.A. (2021). Bitten or struck by dog: A rising number of fatalities in Europe, 1995–2016. Forensic Sci. Int..

[6] Iarussi F., Cipolloni L., Bertozzi G., Sasso L., Ferrara M., Salerno M., Rubino G.T.R., Maglietta F., Dinisi A., Albano D. (2020). Dog-bite-related attacks: A new forensic approach. Forensic Sci. Int..

[7] Tsuji A., Ishiko A., Kimura H., Nurimoto M., Kudo K., Ikeda N. (2008). Unusual death of a baby: A dog attack and confirmation using human and canine STRs. Int. J. Legal Med..

[8] Bonuglia M., Chiara B., Grasso C., Russo A., De Iuliis P. Canine STRs analysis in a forensic casework. Proceedings of the XXXII Conference of the International Society for Animal Genetics (ISAG).

[9] Clarke M., Vandenberg N. (2010). Dog attack: The application of canine DNA profiling in forensic casework. Forensic Sci. Med. Pathol..

[10] Shutler G.G., Gagnon P., Verret G., Kalyn H., Korkosh S., Johnston E., Halverson J. (1999). Removal of a PCR inhibitor and resolution of DNA STR types in mixed human-canine stains from a five year old case. J. Forensic Sci..

[11] El-Shorbagy H.M., El-Liethy S.S., Moussa M.K., Elghor A.A. (2022). Carrier RNA is a key factor affecting fully integrated short tandem repeats profiling in challenging forensic samples models. J. Basic Appl. Zool.

[12] Kunita F., Udagawa C., Inagaki T., Suzuki H., Bonkobara M., Omi T. (2024). Population genetics for 18 short tandem repeat loci (Canine Genotypes(TM) Panel 2.1 Kit) of 150 unrelated dogs from three pure-bred groups in Japan. Leg. Med..

