# Supplementary material for: A Case of Dog Bite Identification Using Trace DNA Recovered from Clothing Without Apparent Bite Marks
Source: Animals (Basel). 2025 Dec 14;15(24):3587. doi: 10.3390/ani15243587 (PMC12729305; doi:10.3390/ani15243587)
Supplement: Supplementary file 1 [file animals-15-03587-s001.zip › animals-4004876-supplementary.pdf]

Supplementary material

【STR electrophoresis of the trouser sample】

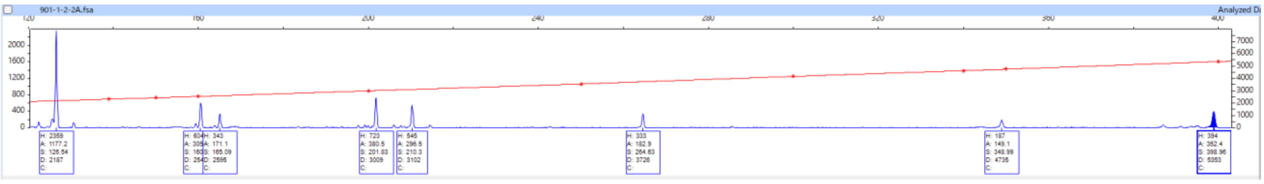

PEZ02 ZFX/Y PEZ17 FH2017 FH2309

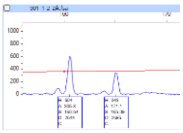

(ZFX/Y)↑overlapping allele values

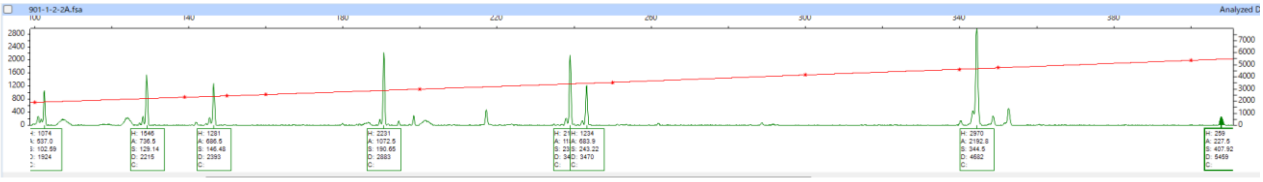

PEZ05 FH2001 FH2328 FH2004 FH2361

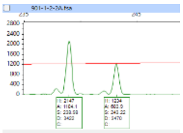

（FH2004）

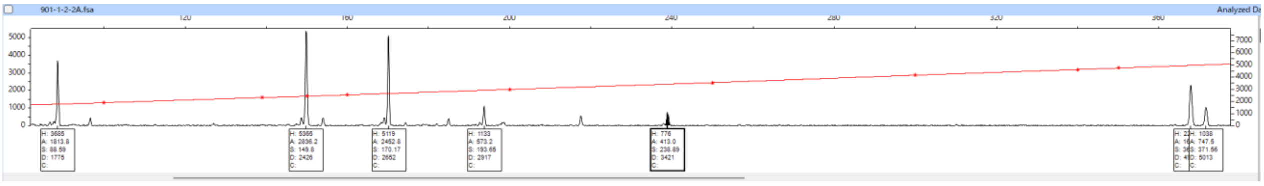

PEZ21 FH2054 FH3377

FH2107

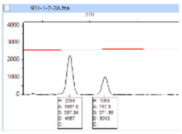

（FH2107）

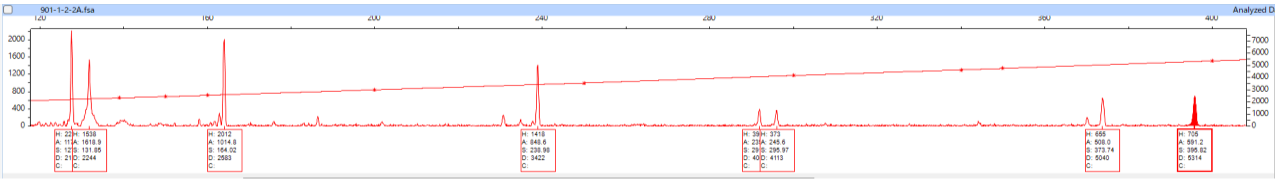

FH2088 vWF.X FH2010 PEZ16

FH3313

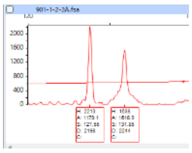

（FH2088）

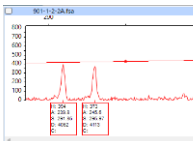

（PEZ16）

---

【STR electropherogram of Candidate Dog 1】

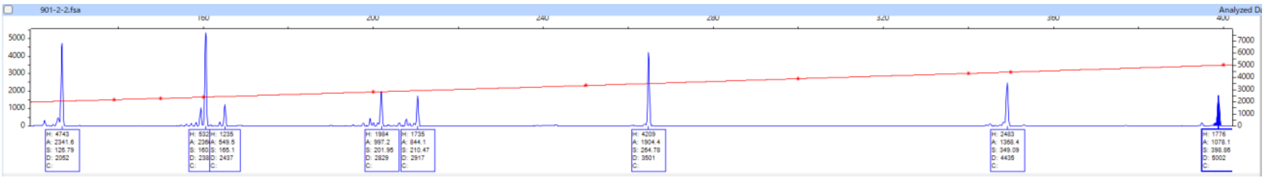

PEZ02 ZFX/Y PEZ17 FH2017 FH2309

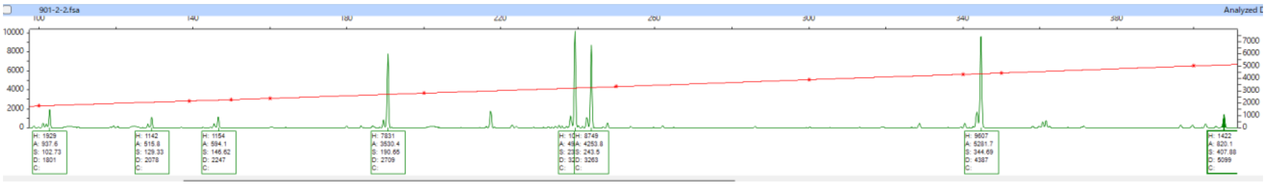

PEZ05 FH2001 FH2328 FH2004 FH2361

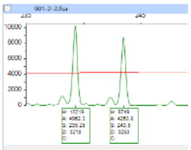

（FH2004）

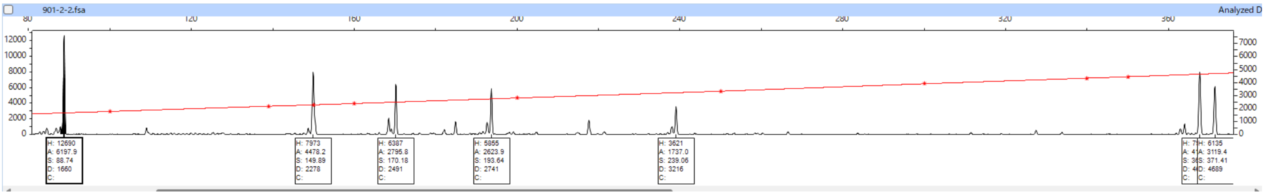

PEZ21 FH2054 FH3377

FH2107

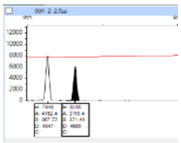

（FH2107）

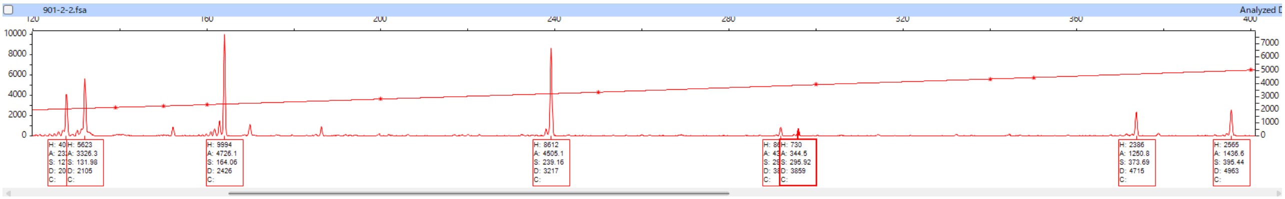

FH2088　　vWF.X　　FH2010　　PEZ16

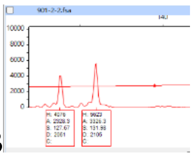

FH3313

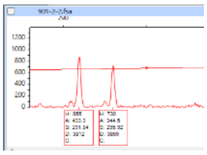

(FH2088)　　（PEZ16）

---

【STR electropherogram of Candidate Dog 2】

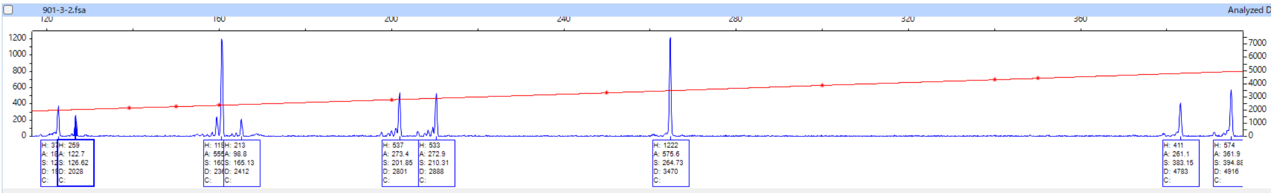

PEZ02　　ZFX/Y　　PEZ17　　FH2017

FH2309

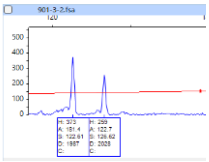

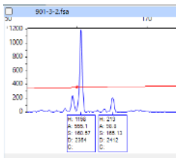

（PEZ02）　　（ZFX/Y）

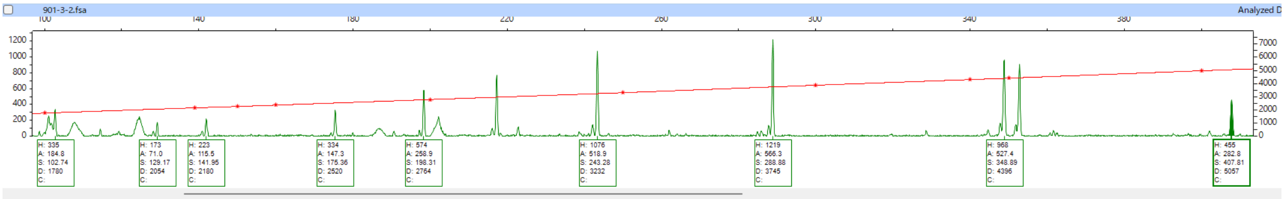

PEZ05 FH2001 FH2328 FH2004 FH2361

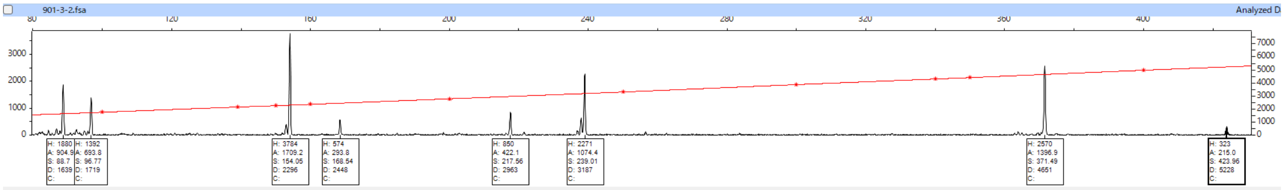

PEZ21 FH2054 FH3377 FH2107

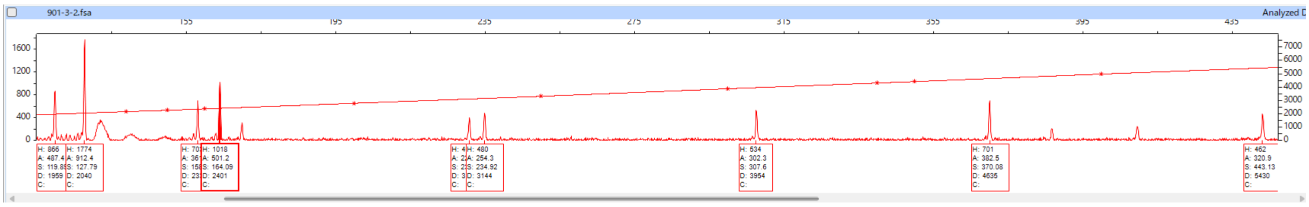

FH2088 vWF.X FH2010 PEZ16 FH3313

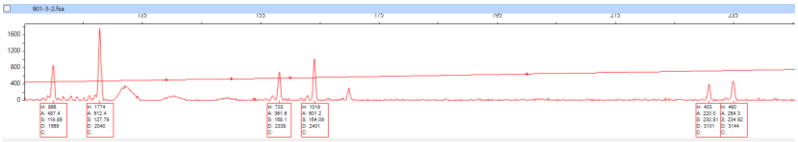

（FH2088 vWF.X FH2010）

---

【STR electropherogram of Candidate Dog 3】

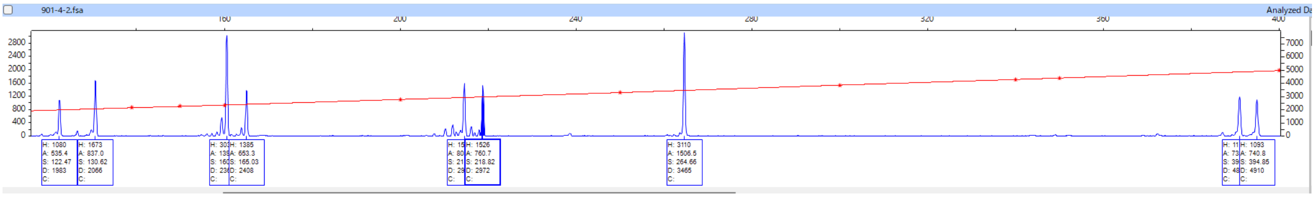

PEZ02 ZFX/Y PEZ17 FH2017

FH2309

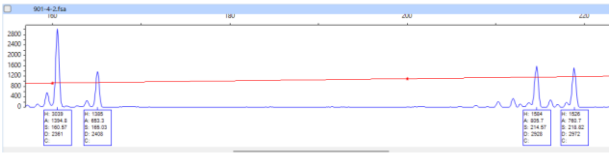

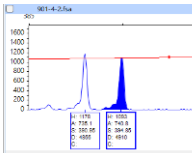

（ZFX/Y PEZ17） （FH2309）

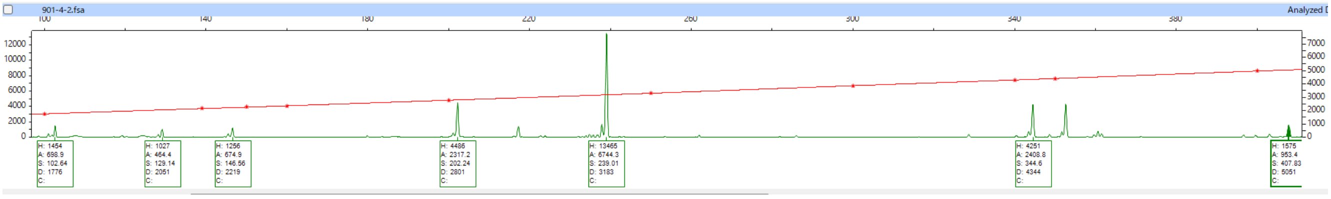

PEZ05 FH2001 FH2328 FH2004

FH2361

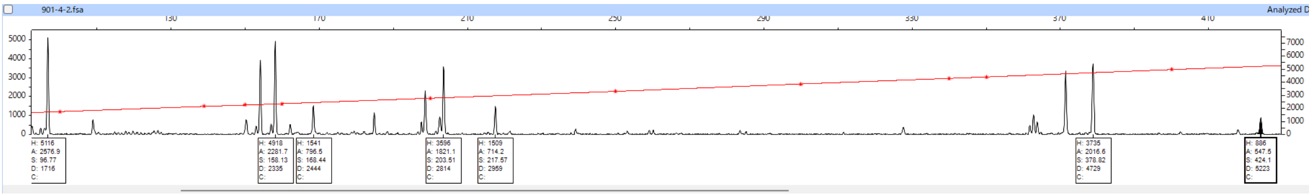

PEZ21 FH2054 FH3377

FH2107

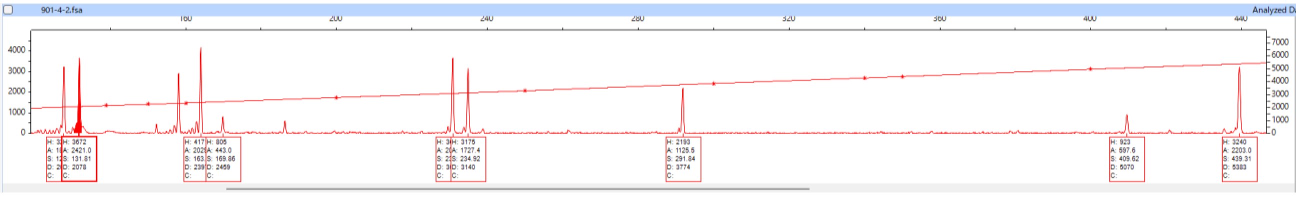

FH2088 vWF.X FH2010 PEZ16

FH3313

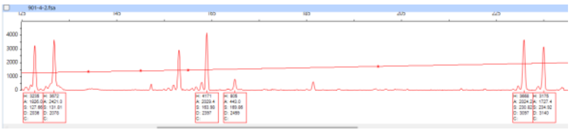

（FH2088 vWF.X FH2010）
